# Integrating Individual-Level Syndromic Surveillance Data Into Infectious Disease Modeling for COVID-19 Forecasting in Sweden: Comparative Modeling Study

**DOI:** 10.2196/104804

**Published:** 2026-10-29

**Authors:** Shomaila Mazhar, Jonas Björk, Dominik Dietler

**Affiliations:** 1 Division of Occupational and Environmental Medicine Lund University Lund, Skåne Sweden; 2 Clinical Studies Sweden, Forum South Skåne University Hospital Lund, Skåne Sweden

**Keywords:** 1177 health care helpline, COVID-19 prediction, epidemiological modeling, individual-level data, syndromic surveillance

## Abstract

**Background:**

The COVID-19 pandemic has triggered major global challenges, highlighting the need for timely surveillance and accurate forecasting to curb disease transmission and reduce the health burden. Traditional modeling approaches usually rely on aggregated data on confirmed cases. However, testing data can be unreliable due to low availability and uptake, as well as registration delays. In Sweden, the 1177 health care helpline provides valuable syndromic surveillance data that can capture early signals of infection prior to confirmation through testing.

**Objective:**

This study aims to evaluate the effect of integrating syndromic surveillance data on short-term COVID-19 forecast accuracy.

**Methods:**

We developed a novel epidemiological model named SI_s_I_c_HRD (susceptible-infected_suspected_-infected_confirmed_ -hospitalized-recovered-deceased), incorporating data from the 1177 health care helpline cross-linked with national registers on COVID-19 testing, hospitalizations, and deaths. In the novel model, the population with infection is divided into suspected infections (I_s_; those reporting symptoms to 1177) and confirmed infections (I_c_; those who tested positive). Accuracy metrics of this model are compared to a model without data from the 1177 health care helpline. To evaluate model performance across different pandemic phases between March 2 and December 31, 2020, we used a daily rolling-window approach with a 60-day fitting period to predict confirmed infections, hospitalizations, and deaths over the subsequent 30 days. Prediction performance was evaluated using mean absolute error and mean absolute percentage error (MAPE).

**Results:**

The proposed SI_s_I_c_HRD model successfully captured the temporal dynamics of confirmed, hospitalized, and death cases. The model achieved a median mean MAPE of 61.6% (IQR 37.6%-79.6%) for confirmed cases, 37.5% (IQR 18.9%-57.5%) for hospitalizations and for deaths, the model achieved a median MAPE of 0.65% (IQR 0.3%-1.9%), indicating substantially higher predictive accuracy for severe outcomes compared to confirmed infections and hospital admissions. Compared to the model without syndromic surveillance data, short-term forecasting performance of the SI_s_I_c_HRD model reduced MAPE by an average of 15.7 percentage points for confirmed cases, 8.17 percentage points for hospitalizations, and 1.99 percentage points for deaths. Overall, the results demonstrate predictive stability for severe outcomes during the early phase of the pandemic.

**Conclusions:**

The proposed model demonstrated predictive performance by integrating individual-level data with health care helpline syndromic data on testing and severe disease outcomes, capturing early signals from symptomatic individuals and distinguishing suspected cases from subsequently confirmed infections, leading to better forecasts of severe outcomes such as hospitalizations and deaths. This framework can support public health decision-making in future outbreaks.

## Introduction

Pandemics have repeatedly caused significant loss of life and triggered severe economic crises worldwide [1]. In 2019, the world encountered one of the deadliest pandemics in modern history, the SARS-CoV-2, also known as COVID-19, which resulted in approximately 18.2 million deaths worldwide from January 1, 2020, to December 31, 2021 [2]. SARS-CoV-2 belongs to the coronavirus family and is primarily transmitted from human to human. Among coronaviruses, it is considered the most contagious [3]. Due to its rapid global spread, the World Health Organization officially declared it a global pandemic on March 11, 2020 [4]. In Sweden, the first COVID-19 case was reported on January 31, 2020, involving a traveler who had arrived from Wuhan, China [5]. By mid-March 2020, the virus had spread across all regions of Sweden, posing significant challenges for public health surveillance and health care systems [6,7]

Effective monitoring of community-level disease transmission is crucial for public health and health care systems [8]. To predict and monitor disease transmission, evaluate the course of the pandemic, and assess the impact of public health intervention policies, epidemiological mathematical models are often used [9]. Such models have proven to be valuable tools for policymakers, enabling data-driven decision-making by exploring different scenarios and their potential outcomes in a controlled manner [10,11]. Among these, the susceptible-infected-recovered (SIR) model is widely regarded as one of the most fundamental and influential frameworks for describing the transmission dynamics of infectious diseases [12]. Over time, several extensions of the SIR model, including susceptible-infected-recovered-susceptible, susceptible-exposed -infected-recovered, and susceptible-infected-susceptible, have been developed to improve short-term infection forecasting [13-15].

While various approaches exist, much of the focus has traditionally been on confirmed cases identified through widespread testing programs. However, traditional case counts are often insufficient for early detection of novel infectious diseases due to limitations in testing capacity and laboratory resources [6]. Syndromic surveillance offers an effective alternative, providing a more comprehensive view of disease dynamics [16-18]. For instance, in the United States, researchers developed a smartphone-based syndromic surveillance system and identified loss of taste and smell as strong predictors of COVID-19 infection [16]. Similarly, app-based self-reported symptoms data have been used to predict COVID-19 cases in Sweden, England, and the United Kingdom [19-23]. Recent studies have indicated that integrating data-driven and epidemiological models, when combined with syndromic and digital data sources, can improve the rapid detection and prediction of infectious diseases. Mathematical and computational models have not only guided immediate responses but also informed medium- and long-term strategies, playing a crucial role in policy planning, public health interventions, and global coordination during the pandemic [24]. Similarly, in a study conducted in 2021, the authors used time series of online search query frequencies to gain insights into COVID-19 prevalence across multiple countries [25]. In these studies [26,27], the authors extended classical compartmental models by incorporating media coverage as a behavioral and informational factor influencing disease transmission dynamics. Drew et al [28] introduced a COVID-19 Symptom Study app in 2020 to collect large-scale, real-time data on symptoms, risk factors, and outcomes, which was subsequently used for scalable epidemiological analysis and modeling to inform public health response.

To respond effectively to outbreak events, policymakers must be well-prepared, relying on real-time and historical data collected from epidemiological, health care, and social processes [29-32]. In Sweden, syndromic surveillance has become a valuable complement to traditional public health monitoring, offering real-time insights into the spread of COVID-19 and other infectious diseases [19]. One important source of syndromic data is Sweden’s National Telehealth Service, the 1177 health care helpline, which often serves as a first point of contact with the health care system for patients. While data are captured for administrative purposes, they have been used in several epidemiological studies for early detection of water-borne disease outbreaks and during the COVID-19 pandemic [33-35]. For example, Spreco et al [34] used aggregated 1177 data as an early signal to estimate potential health care demands in the upcoming weeks. Moreover, a study conducted in 2024 found that calls to Sweden’s 1177 health care helpline reporting COVID-19–related symptoms were important predictors of hospitalizations during the early phase of the pandemic [35].

While previous studies highlight the potential of syndromic surveillance data for disease monitoring, their added value when linked to comprehensive individual-level health register information for improving model accuracy remains poorly understood. In contrast to previous approaches based primarily on aggregated syndromic surveillance data, our study integrates individual-level 1177 symptom data with linked health register information within a compartmental transmission model, allowing individuals to be followed from symptom reporting through subsequent testing and severe disease outcomes. Therefore, in this paper, we extend traditional mathematical models to incorporate a compartment for suspected infection based on individual-level syndromic surveillance data linked with health register data. This approach enables the model to capture early symptomatic signals at the individual level and connect them with subsequent testing outcomes and severe disease progression. The aim of this study is to assess the effect of this integration on the accuracy of short-term infectious disease predictions using a model without syndromic surveillance data as a benchmark.

## Methods

### Overview

The study population consists of all individuals registered in Sweden on December 31, 2019. The first year of the COVID-19 pandemic was analyzed, covering the period from March 2 to December 31, 2020. Daily confirmed infections, hospitalizations, and deaths were incorporated to capture the temporal dynamics of the disease. This section outlines the data sources, model formulation, and discusses the process for tracing the flow of individuals through the different model compartments using individual-level data.

### Data Sources

Information on key symptoms, including fever, cough, and shortness of breath, was extracted from 1177 records and used as the basis for defining suspected infections in the modeling framework. Telehealth call data were obtained from the Swedish 1177 health care advice helpline. During the COVID-19 outbreak in 2020, the number of people calling the helpline for advice on COVID-19–related symptoms spiked, providing valuable syndromic surveillance information [33]. Individual-level demographic data were retrieved from the Swedish Total Population Register. Information on laboratory-confirmed cases was obtained from the national register on notifiable diseases, SMINet, maintained by the Public Health Agency of Sweden. Data on hospital admissions for COVID-19 were extracted from the National Patient Register, while dates of death were obtained from the Swedish Tax Authority. Individual-level data from the 1177 health care helpline were linked with multiple national register-based data sources using the personal identification number assigned to all Swedish residents at birth or immigration. This linkage enables tracking of individuals from symptom reporting to testing outcomes, hospitalization, death, or recovery.

### Epidemiological Model

In this study, we developed 2 epidemiological models. The first is a baseline model, referred to as the SI_c_HRD (susceptible-infected_confirmed_-hospitalized-recovered-deceased) model. The second is an extended version of this framework that integrates data from the 1177 health care helpline, termed the SI_s_I_c_HRD (susceptible-infected_suspected_-infected_confirmed_ -hospitalized-recovered-deceased) model. The only difference between the models is the addition of the I_s_ compartment that integrates the 1177 symptom data, while all other compartments and transitions remain unchanged in Figure 1. No births or natural deaths were included in the model due to the relatively short study period.

**Figure 1:**
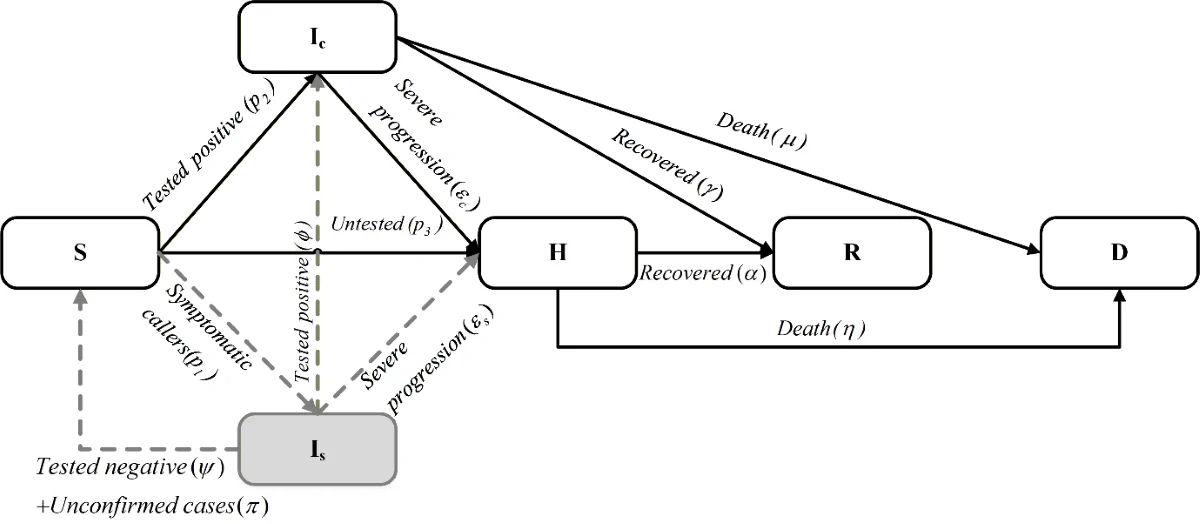
Block diagram of the SI_s_I_c_HRD (susceptible [S]-infectedsuspected [Is]-infectedconfirmed [Ic]-hospitalized [H]-recovered [R]-deceased [D]) model.

### SI_c_HRD Model

This baseline model divides the population into 5 compartments: susceptible (S), infected_confirmed_ (I_c_), hospitalized (H), recovered (R), and deceased (D). The corresponding system of differential equations governing the dynamics of each compartment is given below. The model parameters along with their descriptions are summarized in Table 1.



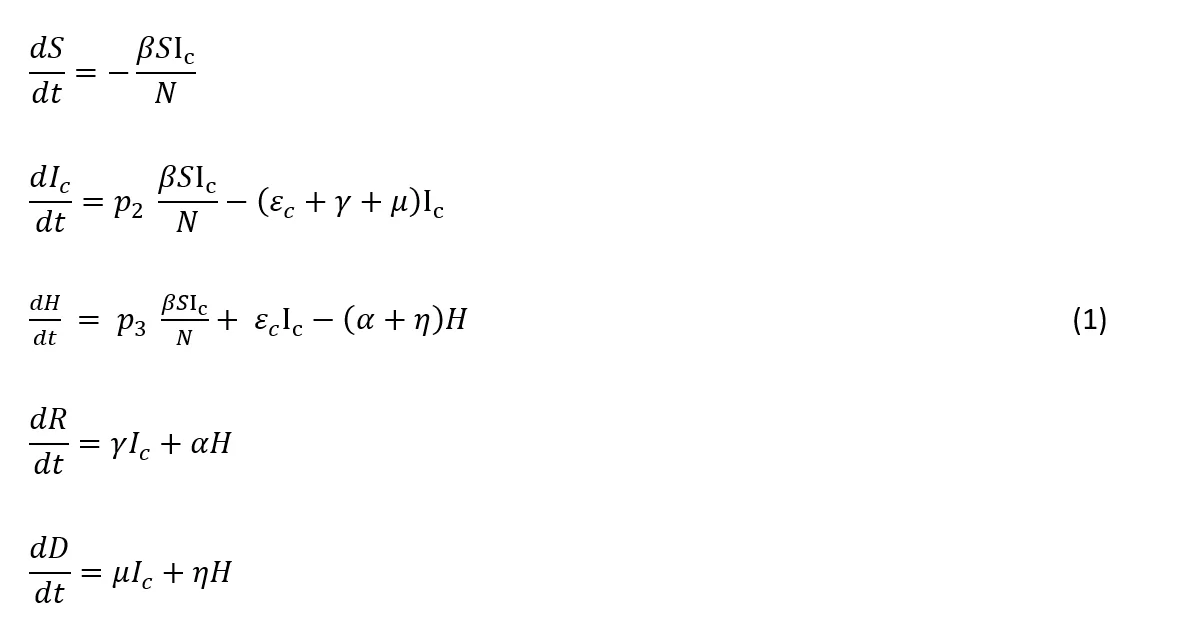



Here, *β* denotes the transmission rate, *p*_2_ represents the probability of an individual becoming infected, and *ε_c_* corresponds to the proportion of individuals who develop severe symptoms requiring hospitalization. *γ* is the recovery rate for individuals with infection, while *μ* denotes the rate of death among nonhospitalized cases. *p*_3_ represents the probability that an individual is directly admitted to the hospital without prior testing, *α* is the recovery rate for hospitalized patients, and *η* indicates the rate of death within the hospital.

**Table 1:** Model parameters and their descriptions.

Symbols	Description
*β*_*s*_ & *β*_*c*_	Transmission rate of I_s_ & I_c_ compartments
*p_1_*	Probability that a susceptible individual calls and reports COVID-19–related symptoms to the 1177 health care helpline.
*p_2_*	Probability that an individual with infection tested positive without calling 1177
*p_3_*	Probability that an individual with infection is directly hospitalized
*ψ*	Individuals who report COVID-19–like symptoms to the 1177 health care helpline but are subsequently tested negative for COVID-19.
π	Return to S (if not in I_c_ or H within 14 days)
*ϕ*	Transition of individuals who reported symptoms and tested positive
*ε_s_*	Rate of severe progression from I_s_ to H compartment
*ε_c_*	Rate of severe progression from I_c_ to H compartment
*γ*, *α*	Recovery rates from I_c_ & H compartments
*μ*, *η*	COVID-19–related death rates in I_c_ & H compartments

### SI_s_I_c_HRD Model

To incorporate early symptom reporting and detect untested cases, the SI_c_HRD model extends by including individual-level 1177 health care helpline data. In the proposed model, the infected compartment is divided into 2 subgroups, I_s_ and I_c_, resulting in the SI_s_I_c_HRD model.

Individuals in the susceptible compartment may become infected upon contact with infectious individuals, who can be either I_c_ or I_s_. The transmission rates differ for these 2 categories, as some suspected cases may not be infected. *β_c_* denotes the transmission rate from confirmed individuals, while *β_s_* represents the transmission rate from suspected individuals. The rate of change of the susceptible compartment over time is given by:



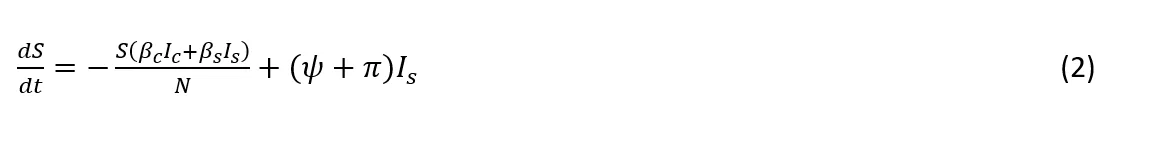



The I_s_ compartment represents individuals who reported their symptoms to the 1177 health care helpline. The probability *p*_1_ represents the fraction of individuals with infection who call and report COVID-19 symptoms before any positive test or hospital admission enters the I_s_ compartment. Individuals remain in this compartment for 14 days and return to S at a rate π unless they are tested positive (transition to I_c_ at a rate *ϕ*), are hospitalized (transition to H at a rate *ε_s_*), or are tested negative (return to S at a rate *ψ*). The rate of change of the I_s_ compartment over time is given by:



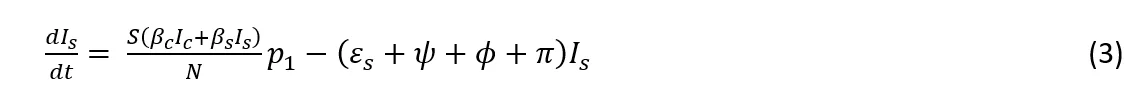



The dynamics of the I_c_ individuals change over time as some newly people with infection test positive for COVID-19. This includes 2 groups: individuals who tested positive without contacting the 1177 health care helpline (at a rate of *p*_2_), and those who reported their symptoms to the 1177 health care helpline and subsequently tested positive within 14 days (at a rate of *ϕ*). Individuals leave compartment I_c_ at the same rates as in the SI_c_HRD model. The rate of change of the I_c_ compartment over time can be expressed as follows:



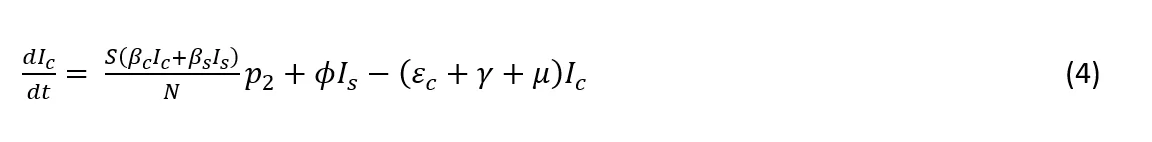



The number of hospitalized individuals increases as severe cases arise from the I_s_ and I_c_ compartments at rates *ε_s_* and *ε_c_*, respectively. Additionally, *p*_3_ represents the probability that the fraction of individuals with infection neither called the 1177 health care helpline nor tested positive and directly required hospitalization. Hospitalized patients leave this compartment either by recovering at a rate *α* or by dying due to COVID-19 at a rate *η*, consistent with the SI_c_HRD model assumptions. The rate of change of the hospitalized compartment over time can be expressed as follows:



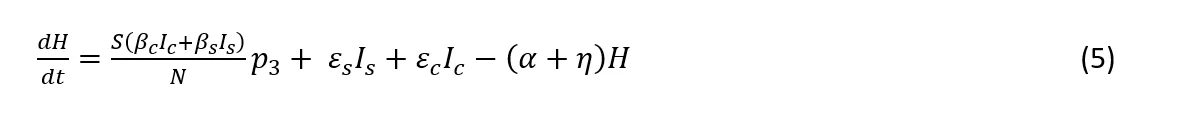



The number of recovered individuals increases at the same rate as in the SI_c_HRD model. The rate at which the recovered compartment changes over time can be expressed as follows:



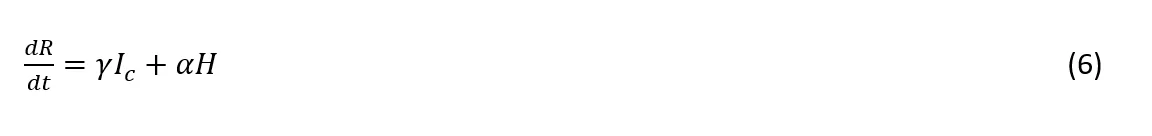



The number of deceased individuals increases as people die due to COVID-19 from the I_c_ compartment at rates *μ*, as well as from the hospitalized compartment at rate *η*. The rate of change of the deceased compartment can be expressed as follows:



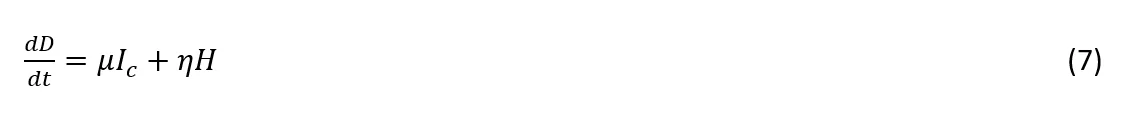



### Parameters Estimation and Model Calibration

To evaluate model performance, the first step involves optimizing model parameters. A total of 14 unknown parameters were estimated using individual-level data, that is,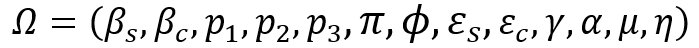. The study population could be directly assigned to their respective compartment at any given time point. Hence, the initial conditions of all compartments were directly extracted from the individual-level data. The stepwise fitting and prediction procedure is described as follows:

A short-term fitting and rolling calibration framework was applied in this study, consistent with the use of moving or sliding-window approaches in previous short-term forecasting studies [34,36]. These studies have used different fitting, evaluation, and prediction periods and step sizes. In this study, a 60-day moving fitting window with a 1-day step size was used. At each prediction date, the model parameters were estimated using the preceding 60 days of observations and subsequently used to generate predictions for the following 30 days. The 60-day fitting window was selected to provide sufficient observations for reliable parameter estimation while maintaining the ability to capture temporal variations in transmission patterns. Random values between 0 and 1 were used as initial parameter values. The parameters were then optimized by solving the system of model equations numerically using MATLAB’s ode15s solver, suitable for stiff equations, while minimizing the objective function that quantifies the discrepancy between observed (actual) *y_i_^obs^* and model predicted values *y_i_^pred^* at each time point *i*, where *n* denotes the total number of days:



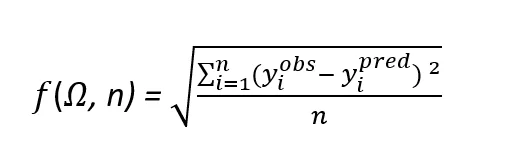



### Model Prediction and Evaluation

The fitted parameters were subsequently used to generate model predictions. After parameter estimation, the model was simulated for the following 30 days, and the predicted values were compared with the observed data for validation. Model performance was assessed using the error metrics mean absolute error (MAE) and mean absolute percentage error (MAPE).



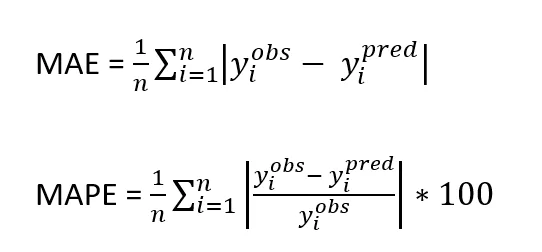



Here, *y_i_^obs^* denotes the observed (actual) number of cases (eg, confirmed infected, hospitalized, and number of deaths actual cases) at time point *i*, and *y_i_^pred^* represents the corresponding model-predicted value. The index *i* = 1,2,...,*n* refers to the total number of observed time points (days). To further evaluate predictive performance, both the baseline model and the extended model incorporating 1177 syndromic surveillance data were fitted using a 60-day rolling window. These fitted models were then used to generate 30-day-ahead predictions for confirmed cases, hospitalizations, and deaths. The study period spans from March 2, 2020, to December 1, 2020. Model calibration and forecasting were performed using a rolling-window approach, with a 60-day fitting window followed by a 30-day prediction window. The fitting window was shifted forward by 1 day for each subsequent calibration. A total of 216 daily error values were analyzed for each compartment over the evaluation period (May 1, 2020, to December 1, 2020). For further analysis, the evaluation period was divided into 3 distinct periods. Period 1 covers (May 1, 2020, to June 10, 2020), period 2 spans (June 11, 2020, to August 31, 2020), and period 3 includes (September 1, 2020, to December 1, 2020).

### Ethical Considerations

This study was reviewed and approved by the Swedish Ethical Review Authority, with original permit number 2020-06492 and subsequent amendments 2021-01115, 2022-01355-02, 2022-06118-02, and 2024-02342-02.

## Results

The number of callers reporting each symptom, together with the corresponding counts of confirmed infections (I_c_), hospitalized, and no confirmed infection, was summarized in Table 2. A substantial number of individuals reporting symptoms were later classified as having no confirmed infection (unconfirmed symptomatic callers with respect to COVID-19). In particular, 312,784 (94.78%) individuals reported breathing difficulties, 468,379 (94.41%) reporting fever, and 326,623 (95.43%) reporting cough were identified as having no confirmed infection. This group includes individuals who tested negative as well as those who were not tested and were not subsequently observed in any other compartment within a 14-day follow-up period. This high number of unconfirmed symptomatic callers may reflect heightened public concern, stress, and anxiety during the pandemic, particularly during the initial phase in March-April 2020 when 1177 call volumes increased sharply. In addition, the limited availability of COVID-19 testing during this early phase may have resulted in symptomatic individuals remaining unconfirmed despite potentially having an infection.

**Table 2:** Frequency of COVID-19–related symptoms reported to the 1177 health care helpline during the study period March-December 2020 and transitions to downstream compartments. Confirmed infections (Ic) include individuals who tested positive within 14 days after symptom reporting. Hospitalized individuals are those who are directly admitted for COVID-19 after symptom reporting without prior testing. Individuals who were not tested positive, hospitalized, or died within 14 days were considered to have no confirmed infection.

Symptoms	Suspected infections (I_s_)	Confirmed infections (I_c_), n (%)	Hospitalized (H), n (%)	No confirmed infection, n (%)
Breathing difficulties	330,066	13,407 (4.06)	3875 (1.17)	312,784 (94.78)
Fever	496,087	23,497 (4.74)	4211 (0.85)	468,379 (94.41)
Cough	342,248	13,749 (4.01)	1876 (0.55)	326,623 (95.43)

The MAE and MAPE of infected confirmed cases (I_c_) for the models with and without 1177 data, along with their difference over time, are presented in Figure 2 (panels A and B). The time series analysis shows clear temporal variation in model performance. Early in period 1, the model incorporating 1177 data generally exhibits lower prediction errors compared to the model without 1177. However, during the summer starting period (period 2), both models show increased error, with relatively higher variability in the model with 1177. Late in period 3 (November-December), the model with 1177 again demonstrates improved performance, with lower error compared to the model without 1177. Specifically, the median MAPE difference was 5.26% (IQR –4.80% to 25.25%) in period 1, –0.07% (IQR –22.96% to 46.22%) in period 2, and 0.0008% (IQR –1.75% to 0.07%) in period 3.

Furthermore, box plot analysis (Figure 2, panels C and D) compares the distribution of MAE and MAPE for both models across the 3 periods. In period 1, the model incorporating 1177 shows a median MAPE of 31.37% (IQR 20.03%-39.96%), compared to 33.33% (IQR 25.73%-58.86%) for the model without 1177. In period 2, the model with 1177 yields a median MAPE of 82.90% (IQR 74.51%-160.5%), compared to 98.13% (IQR 63.82%-168.5%) without 1177. In period 3, the model with 1177 reports a median MAPE of 59.35% (IQR 35.72%-64.11%), whereas the model without 1177 shows a median MAPE of 56.94% (IQR 49.35%-64.12%).

Finally, Figure 2 (panel E) illustrates the fitting and prediction performance of both models. The model incorporating 1177 data achieves a MAE of 488.58, whereas the model without 1177 data shows a substantially higher MAE of 16901.38, indicating poor predictive performance in the absence of helpline data.

**Figure 2:**
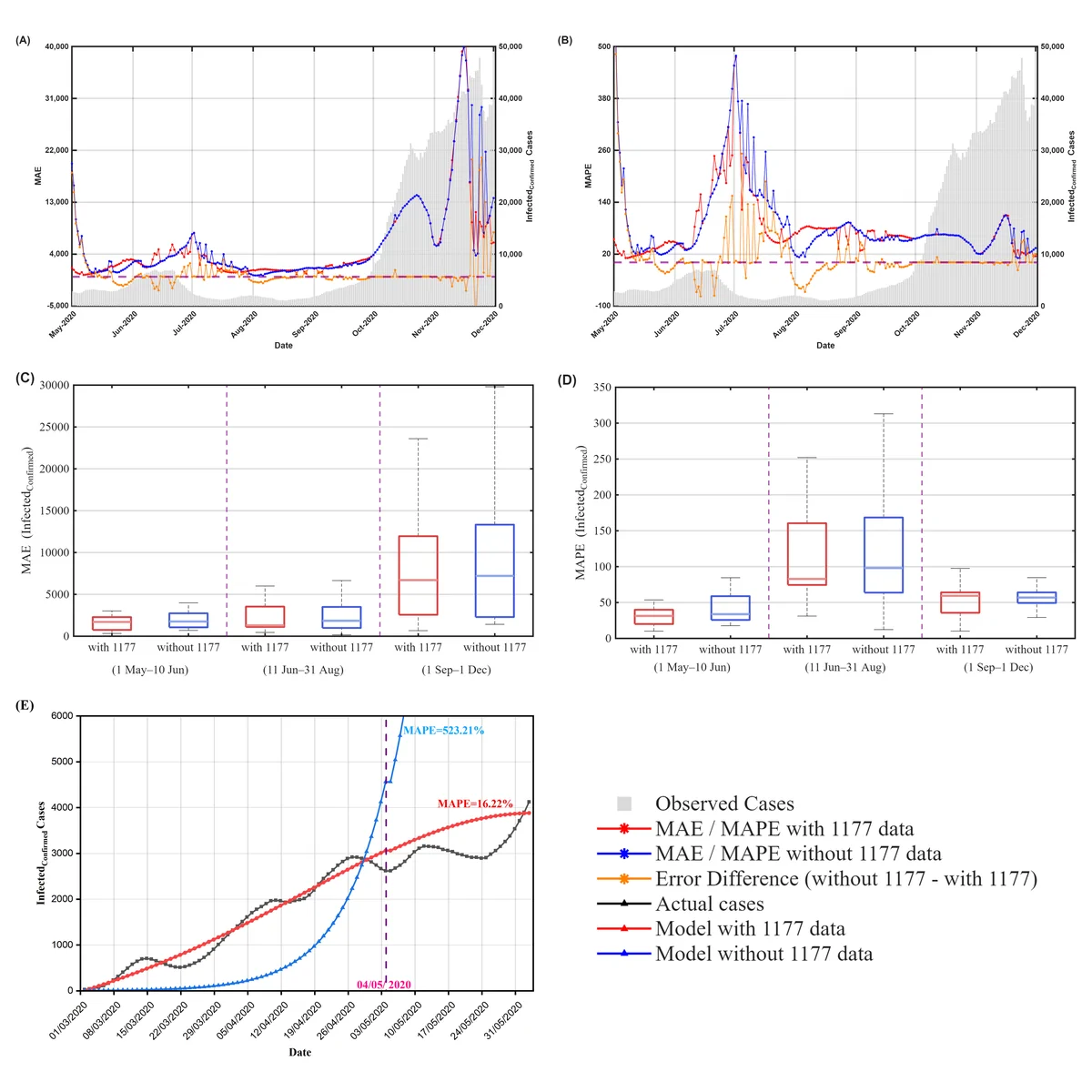
Performance comparison of the SI_c_HRD (susceptible-infected_confirmed_-hospitalized-recovered-deceased; without 1177) and SI_s_I_c_HRD (susceptible-infected_suspected_-infected_confirmed_-hospitalized-recovered-Deceased; with 1177) models for InfectedConfirmed cases. (A-B) The temporal variation of the mean absolute error (MAE) and mean absolute percentage error (MAPE), respectively, for the models with and without 1177 health care helpline data. The blue line represents the SI_c_HRD model (without 1177 data), whereas the red line represents the SI_s_I_c_HRD (with 1177 data). The orange line indicates the difference in error values between the 2 models, highlighting periods where the inclusion of 1177 data improves (positive values) or reduces (negative values) predictive performance. The gray histogram in the background represents the daily number of observed confirmed cases. (C-D) The box plot distributions of MAE and MAPE, respectively, for both models across the 3 evaluation periods. (E) The short-term fitting and 30-day-ahead prediction performance. Black lines represent the observed COVID-19 data in Sweden, blue lines indicate the fitting and prediction results of the SI_c_HRD (without 1177) model, and red lines indicate the results of the SI_s_I_c_HRD (with 1177). The purple dashed line marks the transition between the fitting and prediction periods. The corresponding MAPE values are displayed using the same color as each model curve, enabling direct comparison of predictive performance.

Figure 3 (panels A and B) presents the MAE and MAPE of hospitalized cases (H) for the models with and without 1177 data, along with their temporal difference. The time-series analysis reveals clear variations in model performance across different epidemic periods. Early in period 1, the model incorporating 1177 data generally performs better. However, during the summer period (period 2), both models exhibit poorer performance, with slightly higher errors observed in the model with 1177. In the subsequent period 3, the relative performance alternates, with the model with 1177 showing improved accuracy at certain time points, while the model without 1177 performs better at others. Specifically, the median MAPE difference was 0.0097% (IQR –2.69% to 8.57%) in period 1, 0.42% (IQR –9.38% to 42.24%) in period 2, and –1.27% (IQR –9.08% to 1.19%) in period 3.

Panels C and D in Figure 3 present box plots of MAE and MAPE for both models, enabling a period -wise comparison. In period 1, the model incorporating 1177 achieves a median MAPE of 11.72% (IQR 9.17%-16.05%), compared to 15.73% (IQR 9.06%-35.95%) for the model without 1177. In period 2, the model with 1177 yields a median MAPE of 57.33% (IQR 35.47%-70.77%), which is lower than the 70.73% (IQR 41.68%-98.95%) observed for the model without 1177. In period 3, the model with 1177 reports a median MAPE of 39.44% (IQR 23.60%-53.76%), whereas the model without 1177 shows a slightly lower median MAPE of 33.80% (IQR 17.69%-48.70%).

Finally, panel E in Figure 3 illustrates the fitting and prediction performance for hospitalized cases. The model incorporating 1177 data achieves a MAE of 63.86, compared to 2128.59 for the model without 1177 data, demonstrating that inclusion of 1177 data substantially improves the model’s ability to capture hospital burden, particularly during the early stages of the pandemic.

Figure 4 (panels A and B) presents the MAE and MAPE of deaths (D) for the models with and without 1177 data, along with their temporal differences. The time-series analysis indicates that both models perform well throughout the evaluation period, with the model incorporating 1177 data showing improved performance during period 1. Overall, the difference in MAPE between the 2 models remained small, with the median MAPE difference remaining below 1.20% across the entire evaluation period, suggesting comparable predictive performance for mortality estimation.

Panels C and D in Figure 4 present box plots of MAE and MAPE distributions across the 3 evaluation periods. In period 1, the model incorporating 1177 data achieves a median MAPE of 1.71% (IQR 1.01%-3.93%), compared to 2.93% (IQR 1.48%-22.50%) for the model without 1177 data, indicating improved accuracy when incorporating symptom-reporting information. In period 2, both models demonstrate similar performance, with the model including 1177 data yielding a slightly lower median MAPE of 0.36% (IQR 0.17%-0.69%) compared with 0.38% (IQR 0.07%-0.74%) for the model without 1177 data. In period 3, the model without 1177 data performs marginally better, with a median MAPE of 0.94% (IQR 0.04%-3.07%) compared with 1.06% (IQR 0.28%-3.30%) for the model incorporating 1177 data.

Finally, panel E in Figure 4 illustrates the fitting and prediction performance for deaths. The model incorporating 1177 data achieves a substantially lower MAE of 177.78 compared with 4184.68 for the model without 1177 data, highlighting the benefit of incorporating 1177 information for improving mortality prediction.

Overall, incorporating 1177 health care helpline data improved forecasting performance across all outcomes, including confirmed cases, hospitalizations, and deaths during period 1 of the pandemic. In period 2, the model incorporating 1177 data continued to show slightly better forecasting performance compared with the model without 1177 data, although the overall performance of both models was less favorable than in period 1. In contrast, during period 3, the model without 1177 data performed better than the model incorporating 1177 data, particularly for confirmed cases and hospitalizations. These findings indicate that the forecasting benefit of incorporating 1177 data was most evident in Period 1, remained present but weaker in period 2, and was not consistently observed during period 3.

To provide a comprehensive assessment of model performance, both median errors and average reductions were evaluated over the evaluation period (May 1, 2020, to December 1, 2020). Median MAPE values showed marginal differences between models with and without 1177 data across all outcomes, being 61.6% (IQR 37.6%-79.6%) and 61.4% (IQR 40.6%-91.1%) for confirmed cases, 37.5% (IQR 18.9%-57.5%) and 41.0% (IQR 19.1%-66.0%) for hospitalizations, and 0.65% (IQR 0.3%-1.9%) and 0.74% (IQR 0.3%-2.2%) for deaths. In contrast, the average reductions in MAPE were 15.7, 8.17, and 1.99 percentage points, while the corresponding average reductions in MAE were 685, 53.40, and 101 for confirmed cases, hospitalizations, and deaths, respectively, indicating the greatest improvement for case predictions and confirming overall improved predictive performance following incorporation of 1177 health care helpline data.

**Figure 3:**
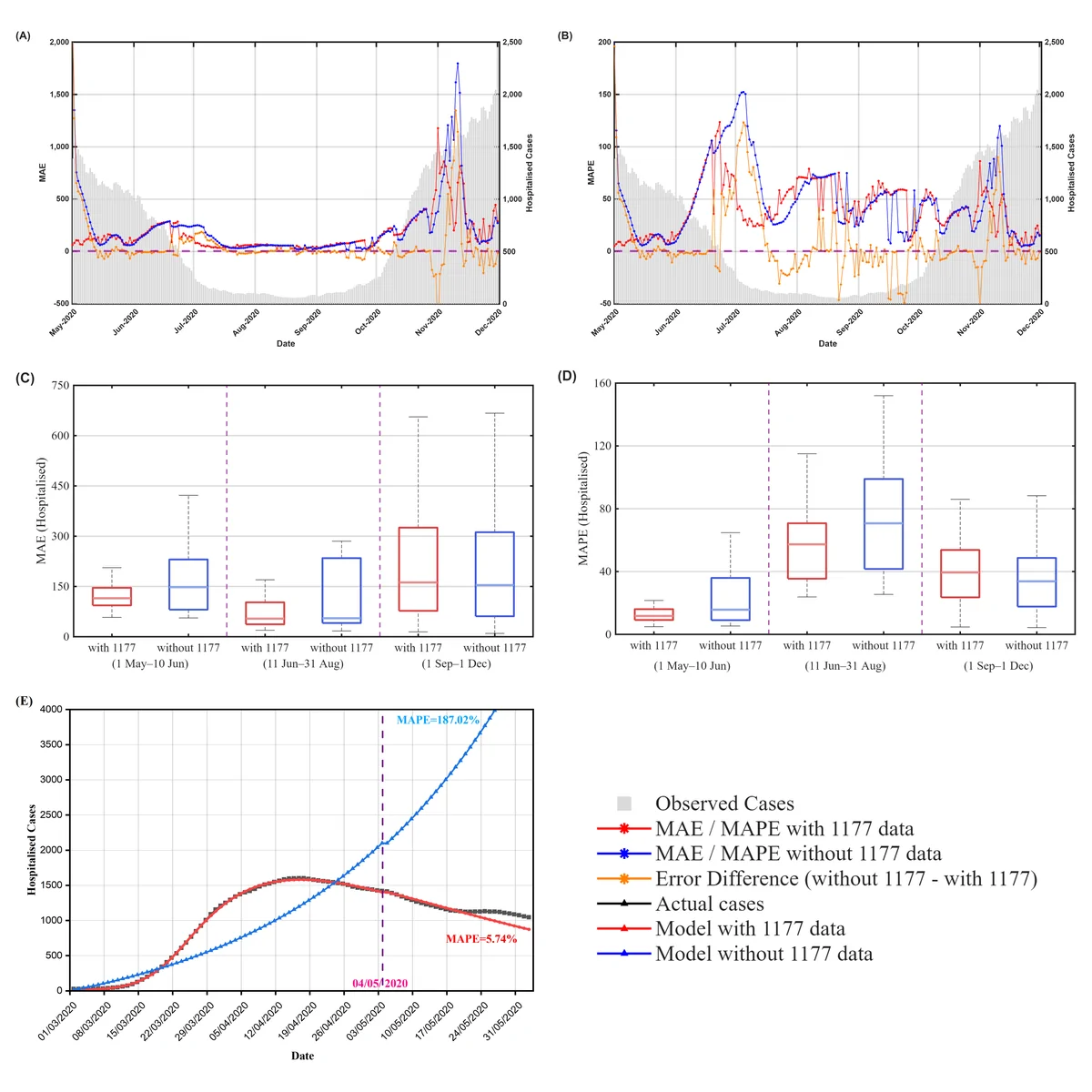
Performance comparison of the SI_c_HRD (susceptible-infected_confirmed_-hospitalized-recovered-deceased; without 1177) and SI_s_I_c_HRD (susceptible-infected_suspected_-infected_confirmed_-hospitalized-recovered-deceased; with 1177), models for hospitalized cases. (A-B) The temporal variation of the mean absolute error (MAE) and mean absolute percentage error (MAPE), respectively, for the models with and without 1177 health care helpline data. The blue line represents the SI_c_HRD model (without 1177 data), whereas the red line represents the SI_s_I_c_HRD (with 1177 data). The orange line indicates the difference in error values between the 2 models, highlighting periods where the inclusion of 1177 data improves (positive values) or reduces (negative values) predictive performance. The gray histogram in the background represents the daily number of observed hospitalized cases. (C-D) The box plot distributions of MAE and MAPE, respectively, for both models across the 3 evaluation periods. (E) The short-term fitting and 30-day-ahead prediction performance. Black lines represent the observed COVID-19 data in Sweden, blue lines indicate the fitting and prediction results of the SI_c_HRD (without 1177) model, and red lines indicate the results of the SI_s_I_c_HRD (with 1177) model. The purple dashed line marks the transition between the fitting and prediction periods. The corresponding MAPE values are displayed using the same color as each model curve, enabling direct comparison of predictive performance.

**Figure 4:**
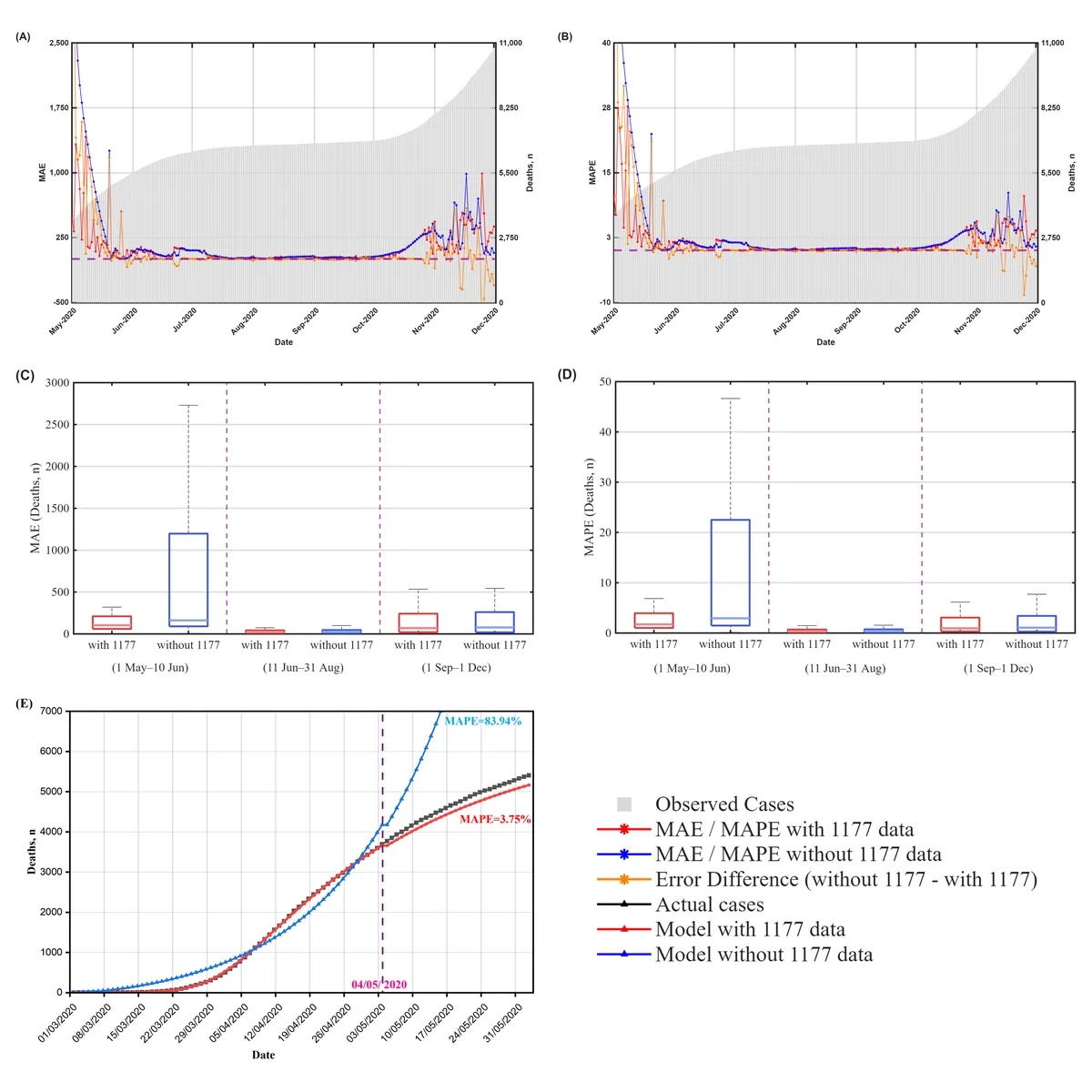
Performance comparison of the SI_c_HRD (susceptible-infected_confirmed_-hospitalized-recovered-deceased; without 1177) and SI_s_I_c_HRD (susceptible-infected_suspected_-infected_confirmed_-hospitalized-recovered-deceased; with 1177) models for the number of deaths. (A-B) The temporal variation of the mean absolute error (MAE) and mean absolute percentage error (MAPE), respectively, for the models with and without 1177 health care helpline data. The blue line represents the SI_c_HRD model (without 1177), whereas the red line represents the SI_s_I_c_HRD (with 1177); the orange line indicates the difference in error values between the 2 models, highlighting periods where the inclusion of 1177 data improves (positive values) or reduces (negative values) predictive performance. The gray histogram in the background represents the number of observed death cases. (C-D) The box plot distributions of MAE and MAPE, respectively, for both models across the 3 evaluation periods. (E) The short-term fitting and 30-day-ahead prediction performance. Black lines represent the observed COVID-19 data in Sweden, blue lines indicate the fitting and prediction results of the SI_c_HRD (without 1177) model, and red lines indicate the results of the SI_s_I_c_HRD (with 1177) model. The purple dashed line marks the transition between the fitting and prediction periods. The corresponding MAPE values are displayed using the same color as each model curve, enabling direct comparison of predictive performance.

## Discussion

In this study, 2 epidemiological models were developed and evaluated: a baseline SI_c_HRD model without 1177 data and an extended SI_s_I_c_HRD model integrating individual-level syndromic surveillance data from the Swedish 1177 health care helpline. The aim was to assess the contribution of health care helpline data to predicting COVID-19 infections. To contextualize model performance, the calling volume data were divided into 3 temporal periods: period 1 (March 2 to June 10, 2020), during which call volumes increased from 19,785 to 48,869; period 2 (June 11 to August 31, 2020), where call volumes gradually decreased from 48,555 to 22,440; and period 3 (September 1 to December 31, 2020), during which call volumes further declined, ranging from 22,515 to approximately 6000. It was observed that higher call volumes during period 1 coincided with lower prediction errors and more stable model performance. The relatively poorer performance observed during period 2 may be partly attributable to the decline in call volume, which reduced the amount of syndromic information available to the model. In addition, changes in health care-seeking behavior during the summer period, including increased travel and social gatherings, may have altered symptom-reporting patterns. The increased availability of COVID-19 testing services may also have reduced the need for individuals to report their symptoms through the 1177 helpline. During period 3, model performance was less favorable, coinciding with a substantial decline in call volume, as fewer individuals reported COVID-19–related symptoms through the 1177 helpline. In addition, the increasing availability of self-testing toward the end of 2020 and greater public awareness of COVID-19 symptoms may have influenced individuals’ decisions to test and seek health care. Overall, the reduced call volume, together with changes in health care-seeking behavior, testing practices, and epidemic dynamics, may have contributed to the reduced forecasting performance observed during periods 2 and 3. Thus, although 1177 data were associated with improved model performance during the earlier periods of the pandemic, their additional predictive value was less evident toward the end of 2020.

Furthermore, the relatively high median MAPE of 61.6% for confirmed cases indicates substantial error in the point forecasts and limits their interpretation as precise estimates of the absolute number of confirmed cases. Several previous studies have reported substantial variation in forecasting accuracy across models, epidemic phases, and forecasting horizons [37,38]. Therefore, the median MAPE observed in our study should be interpreted in the context of the forecasting horizon and the temporal variation in COVID-19 epidemic dynamics.

Incorporating syndromic surveillance data from the 1177 health care helpline improved short-term forecasting performance, reducing MAPE on average by 15.7 percentage points for confirmed cases, 8.2 percentage points for hospitalizations, and 2.0 percentage points for death cases compared to the model without 1177 data. The model effectively captured early infection dynamics during period 1 and maintained reasonable predictive performance during period 2, whereas its performance declined during period 3. These findings highlight the importance of syndromic surveillance data, particularly during the initial phase of a pandemic.

Syndromic surveillance has previously been used in various contexts, including online search trends, symptom-reporting platforms, and mobile health applications, to support early outbreak detection [28,35]. A study conducted in 2025 found that anomalies detected in 1177 health care helpline calls occurred 7 days before confirmed COVID-19 cases, 13 days before hospitalizations, and 22 days before deaths in Sweden [33]. These findings further support the role of helpline-based syndromic data as an early warning signal for emerging outbreaks. In another study, the authors reviewed syndromic surveillance approaches by examining 4 main data streams: emergency department and hospital records, pharmacy and over-the-counter sales, citizen-generated participatory data, and hybrid multisource systems [39]. Their analysis demonstrated that syndromic indicators could detect outbreaks 2-14 days earlier than conventional laboratory reporting, highlighting their potential for timely epidemic detection.

Previous studies have demonstrated the importance of telehealth-based syndromic data for monitoring infectious disease dynamics. In particular, Spreco et al [34] showed that aggregated 1177 health care helpline symptom data could be used for nowcasting COVID-19 trends. They reported satisfactory nowcasting performance for COVID-19 hospitalizations based on reported adult cough symptoms during the high transmission period (April 5-April 25, 2020), with MAPE ranging from 3%-9%, whereas fever-based nowcasting showed substantially higher errors (14%-52%) during the same period. Their approach relied on analyzing temporal trends in aggregated symptom data. In contrast, our study integrates individual-level 1177 symptom to trace individuals across disease states, enabling direct observation of their trajectories through the different compartments. This allows us to assess what happens following symptom reporting, specifically, whether individuals are subsequently confirmed by testing positive, thereby providing real-world evidence on the predictive value of syndromic data. Notably, during period 1 (May 1 to June 10, 2020), the model incorporating 1177 data achieved a median MAPE of 31.37% for confirmed cases. For hospitalizations, the median MAPE was 11.72%, indicating improved predictive performance compared to confirmed cases. For deaths, the model performed best, with a median MAPE of 1.71% over the same period.

A key strength of our study is that the proposed model can predict confirmed COVID-19 cases, hospitalizations, and deaths during the early stage of the pandemic, when call volumes to the 1177 health care helpline were exceptionally high due to heightened public concern and anxiety. The baseline SI_c_HRD model (without 1177 data) showed relatively poor performance during the initial period of the pandemic, likely due to limited testing capacity and reporting delays, which emphasizes the importance of complementary syndromic surveillance data during emerging outbreaks. Incorporating individual-level 1177 symptom data allowed the model to distinguish between laboratory-confirmed cases, unconfirmed symptomatic callers, and hospital cases, thereby reducing predictive errors. These findings highlight that the relative performance of compartmental models and complementary surveillance approaches may vary across different phases of an epidemic or pandemic. During the early phase of an emerging outbreak, when transmission patterns, testing practices, and health care-seeking behavior may change rapidly, syndromic surveillance data may provide useful complementary information when routine surveillance systems are still developing. As the epidemic progresses and surveillance and testing systems become more established, conventional compartmental models may provide useful forecasting information, while the additional contribution of syndromic surveillance may become less evident.

The limitations of this study include reliance on 1177 symptom data, which does not capture individuals who did not contact the service and may therefore miss a substantial number of infections. Although we could follow individuals after symptom reporting using register data, we could not distinguish between untested positive cases and calls related to non-COVID-19–related health issues. Another limitation of this study is that 13 parameters were estimated simultaneously from 3 observed time series. Structural and practical identifiability of the estimated parameters were not formally assessed [40]. Therefore, the uniqueness of the parameter estimates and their reliability under the available data cannot be formally established from the current analysis. Additional factors likely contributing to higher errors include testing delays and underreporting, as well as changes in social behaviors in light of public health recommendations that abruptly altered transmission patterns. Additionally, the use of a daily rolling window may introduce inaccuracies during weekends and holidays. The visualized error trends further highlight the advantage of integrating syndromic surveillance data into infectious disease modeling. Although the baseline SI_c_HRD model performed reasonably well during stable transmission periods, its simplified formulation limited its ability to capture complex epidemic dynamics, particularly during periods of rapid trend changes or multiple waves. The fitted curves often followed smooth exponential-like patterns, reflecting the absence of early symptomatic signals and reduced structural flexibility, which made the model less stable during abrupt shifts in transmission.

Future work could focus on expanding the model by integrating additional data streams, such as mobility patterns, demographic characteristics, vaccination information, wastewater surveillance, and internet search trends, to further improve forecasting accuracy while also accounting for time lags and reporting delays [41-43]. In addition, sensitivity analysis and formal assessments of the structural and practical identifiability of the estimated parameters should be conducted to evaluate the robustness and reliability of the model parameter estimates.

Overall, integrating individual-level syndromic data from the 1177 health care helpline improved short-term COVID-19 forecasting performance during the early stage of the pandemic. The extended model captured early changes in transmission and provided more accurate predictions of confirmed cases, hospitalizations, and deaths during this period. These findings highlight the value of incorporating high-resolution syndromic surveillance data into epidemiological modeling frameworks, while emphasizing the need to account for reporting structures and context-specific data limitations. Although this study uses the Swedish 1177 health care helpline, the proposed framework could be adapted to other health care systems with accessible telehealth or syndromic surveillance data. However, its effectiveness and generalizability may depend on differences in health care-seeking behavior, reporting practices, data availability, and telehealth infrastructure across settings.

## Abbreviations

MAE: mean absolute error
MAPE: mean absolute percentage error
SI_c_HRD: susceptible-infected_confirmed_-hospitalized-recovered-deceased
SIR: susceptible-infected-recovered
SI_s_I_c_HRD: susceptible-infected_suspected_-infected_confirmed_-hospitalized-recovered-deceased

## Data Availability

The data used in this study consist of individual-level records from the Swedish 1177 health care helpline, integrated with national health register data. Due to ethical and legal restrictions under the SWECOV (Swedish Register-Based Research Program on COVID-19) project, these data are not publicly available. Researchers may request access after approval by the Swedish Ethical Review Authority and the respective data providers.
